# Are we ready for *Taenia solium* cysticercosis elimination in sub-Saharan Africa?

**DOI:** 10.1017/S0031182016000500

**Published:** 2016-04-20

**Authors:** MARIA VANG JOHANSEN, CHIARA TREVISAN, SARAH GABRIËL, PASCAL MAGNUSSEN, UFFE CHRISTIAN BRAAE

**Affiliations:** 1 Section for Parasitology and Aquatic Diseases, Department of Veterinary Disease Biology, Faculty of Health and Medical Sciences, University of Copenhagen, Dyrlægevej 100, DK-1870 Frederiksberg C, Denmark; 2 Department of Biomedical Sciences, Institute of Tropical Medicine, Antwerp, Belgium

**Keywords:** *Taenia solium* taeniosis/cysticercosis, intervention tools, control, elimination, sub-Saharan Africa

## Abstract

The World Health Organization announced in November 2014 at the fourth international meeting on ‘the control of neglected zoonotic diseases – from advocacy to action’, that intervention tools for eliminating *Taenia solium* taeniosis/cysticercosis (TSTC) are in place. The aim of this work was to elucidate theoretical outcomes of various control options suggested for TSTC elimination in sub-Saharan Africa (SSA) over a 4-year period. Our current knowledge regarding *T. solium* epidemiology and control primarily builds on studies from Latin America. A simple transmission model – built on data from Latin America – has been used to predict the effect of various interventions such as mass treatment of humans, vaccination and treatment of pigs, and health education of communities, potentially leading to change in bad practices and reducing transmission risks. Based on simulations of the transmission model, even a 4-year integrated One Health approach fails to eliminate TSTC from a small community and in all simulations, the prevalence of human taeniosis and porcine cysticercosis start to rise as soon as the programmes end. Our current knowledge regarding transmission and burden of TSTC in SSA is scarce and while claiming to be tool ready, the selection of diagnostic and surveillance tools, as well as the algorithms and stepwise approaches for control and elimination of TSTC remain major challenges.

## INTRODUCTION

*Taenia solium* is a neglected zoonotic parasite causing epilepsy and severe headaches in humans and substantial economic losses to pig farmers in endemic areas. It has been ranked highest on the global scale of foodborne parasitoses by the Food and Agriculture Organization (FAO) and the World Health Organization (WHO), (FAO and WHO, 2014). WHO considers *Taenia solium* taeniosis/cysticercosis (TSTC) an eradicable disease based on its simple life cycle and availability of powerful and inexpensive disease control tools. The disease has recently been listed as a priority disease for international attention towards elimination, to alleviate poverty (WHO, 2013). Cysticercosis is also one of four neglected zoonoses that are now being targeted for control, elimination, and possibly eradication, as confirmed by the World Health Assembly with the adoption of resolution WHA66·12 on 23 May 2013.

In 2013, it was decided by the WHO to initiate TSTC elimination programmes in selected endemic countries (WHO, 2013). These programmes are expected to run from 2016 to 2020 (WHO, 2015). However, despite being considered eradicable, to date, no endemic country has achieved country level elimination of *T. solium.* Several reasons might exist for the lack of success. Although internationally recognised and declared eradicable, this zoonotic disease remains in most endemic countries – if recognised – trapped in discussions between medical and veterinary responsibilities, like many other zoonotic diseases. In addition, a major constraint is the lack of affordable, easy to apply, sensitive and specific diagnostic tools available for the detection of the various presentations of this parasite. For human taeniosis, Kato smears have mainly been used despite this technique's very low sensitivity and specificity (only providing information about presence of ‘*Taenia* eggs’). Severe under-diagnosis and misdiagnosis of human cysticercosis has also been highlighted. Katabarwa *et al.* (2008) reported several cases of onchocerciasis as misdiagnosed *T. solium* cysticercosis in Uganda. For porcine cysticercosis, the applied diagnostic methods, i.e. tongue palpation and meat inspection, significantly underestimated the true prevalence (Phiri *et al.* 2003; Dorny *et al.* 2004). Braae *et al.* (2015*c*) mapped TSTC in Africa from 1985 to 2014 and found only 141 reports from 476 districts in 29 countries, which clearly shows how neglected this zoonosis is.

Being primarily a socially determined disease, knowledge about its local epidemiology and transmission, remains to be elucidated in many endemic regions (Thys *et al.* 2015). A temporal fluctuation in porcine cysticercosis prevalence was recently reported from Tanzania most likely as a result of farming practices (Braae *et al.* 2014). In the same study, it was also demonstrated that confined pigs had the same level of infection as free-roaming pigs and through a case–control study it was confirmed that feeding potato peels to pigs was a significant risk in getting porcine cysticercosis (Braae *et al.* 2015*a*). Local traditions have also been shown to significantly influence the transmission of *T. solium.* Porphyre *et al.* (2015) found that shortage of pork in connection with festivals, would lead to seasonal variation in trading and slaughtering of infected pork.

The societal burden of TSTC is still unknown due to the lack of epidemiological data. The only parameter included in the DALY calculations for TSTC in humans is epilepsy, despite the knowledge, that this disease causes a number of disabilities. Included in the burden assessment should also be the economic consequences for affected people and pig owners, who lose 50–100% of the value of their pigs if they are infected (Praet *et al.* 2009; Trevisan *et al.* in press).

Nevertheless, in recent years tools for treatment and prevention of TSTC have been developed, providing the hope that the disease can be eliminated if the control tools are applied well. The main tools include treatment of taeniosis cases, treatment of pigs, vaccination of pigs, health education, improved meat inspection, and improved hygiene and sanitation (WHO, 2015; Gabriël *et al*. in press). However, the algorithm (which tools to combine) and the stepwise approach for their application (when to implement which strategy) as well as the goals for success (reduction in presence of the parasite in the final or the intermediate host; reduction in morbidity) and how to measure success remain to be determined.

In this paper, we try to predict the outcome of the different interventions applied in sub-Saharan Africa (SSA), by using a simple Reed-Frost transmission model developed by Kyvsgaard *et al.* (2007).

## PREDICTIONS OF EFFECTS OF INTERVENTIONS

### The simple Reed-Frost model

The model described by Kyvsgaard *et al*. (2007) was modified from a Reed-Frost model developed for infections with bacteria, virus and protozoans. It was developed as both a deterministic and stochastic version where hosts (man and pig) could be susceptible, infected or recovered and presumed immune (only pigs). Transmission between humans and pigs was modelled as susceptible roaming pigs scavenging on human faeces infected with *T. solium* eggs. Transmission from pigs to humans was modelled as susceptible humans eating undercooked pork harbouring *T. solium* cysts. Deterministic models of each scenario were run first followed by stochastic versions of the models to assess the likelihood of infection elimination in the small population modelled. Several assumptions of the model should be mentioned. The model assumes that random contact occurs between hosts, transmission is only direct from man to pig, all infected pigs have equal risk of transmission to man after slaughter, and that the human population is fixed at 1000 individuals. The model was developed based on data from Latin America, but after publication of the model, epidemiological data have been published from SSA, indicating that the basic values of taeniosis (mean prevalence between 1·5 and 5%) and porcine cysticercosis (mean prevalence between 30 and 40%) fitted the model well (Komba *et al.* 2013; Mwanjali *et al.* 2013; Braae *et al.* 2014).

### Treatment of human taeniosis

In the WHO Roadmap (2013) five main intervention strategies were outlined for control of the Neglected Tropical Diseases: preventive chemotherapy [mass drug administration (MDA)], intensified disease management, vector control, improve water quality and sanitation, and zoonotic disease management. Two drugs (praziquantel and niclosamide) are currently registered for taeniosis. Their efficacy is very high although reports have suggested reduced efficacy of niclosamide in some areas (WHO, 2015). Whereas praziquantel is currently available in most TSTC endemic areas, niclosamide is not widely available (WHO, 2015). As MDA of praziquantel [provided annually or biennially (to school-aged children)] is central in control of schistosomiasis, it has been suggested to integrate schistosomiasis and TSTC control in areas of co-endemicity. Major concerns regarding this approach are that TSTC is distributed in clusters, affects all age groups, and treatment of schistosomiasis compared with taeniosis requires four to five times the dose of praziquantel, which may cause severe side-effects in people suffering from neurocysticercosis.

**Fig. 1.**
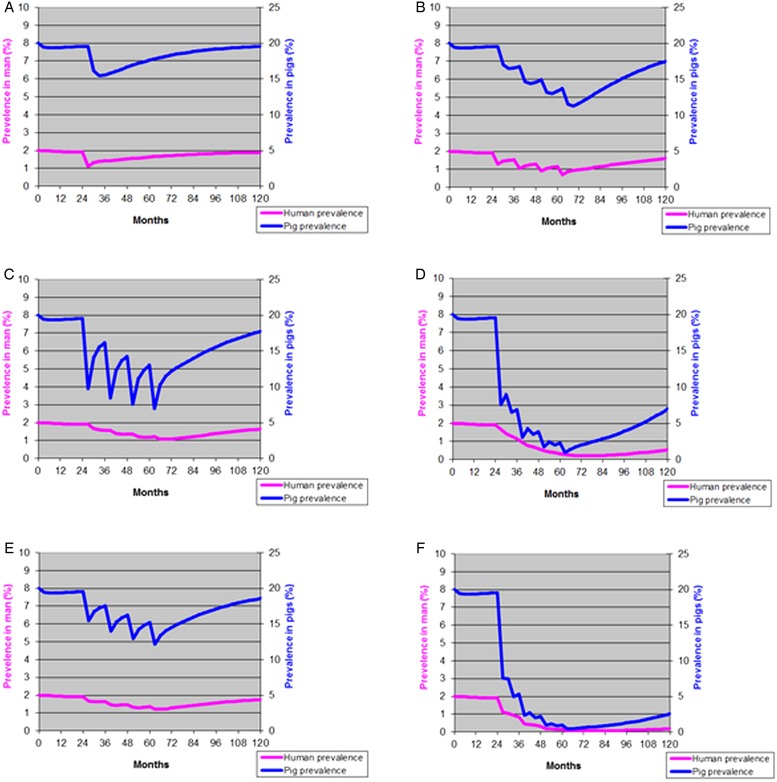
The basic scenario in the model (Kyvsgaard *et al.* 2007) is a small community with 1000 people and 200 pigs (200 families with one pig per family). The prevalence of taeniosis in humans is set at 2% and the prevalence of porcine cysticercosis is 20% based on epidemiological data from Latin America. (A) The effect of a single treatment with praziquantel given to all school-aged children (representing 30% of the population) at month 24 is depicted, assuming a coverage = 100%, and 100% efficacy of the drug. (B) Effect of four treatments with praziquantel, given to all school-aged children (representing 30% of the population) annually for 4 years starting month 24. Coverage = 90%, Efficacy = 100%. (C) Treating 75% of all pigs with oxfendazole (90% efficacious) at yearly intervals for 4 years starting month 24. (D) Vaccination and treatment of 1–9 months old pigs (80% of the pig population) every 6th month for 4 years starting month 24. Coverage 75%, 100% vaccine efficacy and 90% treatment efficacy. (E) Applying health education four times at annual intervals resulting in 50% reduced pig–human and man-to-pig transmission rates due to assumed changed practices in the following 3-month period only. The health education starts from month 24. (F) The scenario depicts the effect of an integrated approach combining (a) four treatments with praziquantel, given to school-aged children (representing 30% of the population) annually for 4 years (coverage = 90%, efficacy = 100%), (b) health education given to the whole population annually for 4 years (50% reduction in transmission rates for 3 months following each intervention) and (c) vaccination and treatment of 1–9 months old pigs given every 6th month for 4 years (80% of the pig population, coverage 75%, 100% vaccine efficacy and 90% treatment efficacy). All interventions start at month 24.

Assuming, in the transmission model, that school-aged children account for 30% of a population, praziquantel at 40 mg kg^−1^ has a 100% efficacy against both mature and immature *T. solium* and coverage is 100%, the model predicts a reduction in taeniosis prevalence from 2 to 1% in the whole population if assessed 3 months later. Similarly, the prevalence of porcine cysticercosis drops from nearly 20 to 15% in the same period (Fig. 1A). If measured a year after the intervention, the reduction is still significant, but as can be seen from the graph, the prevalence of both taeniosis and porcine cysticercosis increases to pre-treatment levels in 4–5 years.

Assuming a similar setting as above but with MDA provided annually for 4 years and a bit more realistic coverage of 90% (Fig. 1B), the prevalence of taeniosis remains about 1% as long as the MDA is ongoing, but starts to increase thereafter. Porcine cysticercosis is generally more affected as the prevalence reduces to about 11%, but increases as soon as the treatment stops.

### Treatment of porcine cysticercosis

Several drugs have been tested on porcine cysticercosis with varying efficacies. Oxfendazole has shown to be the most effective anthelmintic against muscle cysts causing no or little side-effects. However, the drug is not very effective against brain cysts (Mkupasi *et al.* 2013). The drug is not yet registered for treating pigs and not available in most endemic settings. Also, there are no guidelines for evaluating the efficacy of anthelmintics against porcine cysticercosis, and more efficacy studies are needed since the conclusions so far are based on a limited number of studies.

If applying an annual treatment of pigs using oxfendazole with 90% efficacy and 75% coverage, the prevalence of taeniosis would gradually decrease and reach 1% after 3–4 years, but increase as soon as the treatment stops (Fig. 1C). Porcine cysticercosis will significantly decrease right after treatment but with annual intervals, the prevalence remains above 7% and will increase as soon as the treatment stops.

### Vaccination of pigs

Theoretically, a vaccine against porcine cysticercosis with a life-long protection would be the ideal control tool (Garcia *et al.* 2003). At the moment two vaccines have been successfully developed and have shown very high levels of protection in both experimental and field trials (WHO, 2015). The vaccines have not been assessed for cost-effectiveness and are not yet commercially available. Lightowlers (2013) suggested combining vaccination of all pigs between 1 and 9 months of age with oxfendazole treatment, and demonstrated an optimal effect if delivered every 4 months. As such, a high proportion of pigs would have gotten at least two vaccinations and treatments before arriving at slaughter age. Such short interval would however be unrealistically labour-intensive in most settings.

In Fig. 4 Lightowlers' suggestion has been depicted with a 100% efficacious vaccine, a 90% efficacious treatment and coverage of 75% assuming the age group 1–9 months constitutes 80% of the pig population. In this scenario, with the combined intervention every 6 months (depicting two vaccinations per pig in the model reduces taeniosis to <1% and porcine cysticercosis to <5% but returns to pre-treatment levels once the interventions stops.

### Health education

The WHO/FAO/OIE guidelines for prevention and control of TSTC suggest health education to be integrated only as a non-specific measure integrated with other primary health-care messages (WHO/FAO/OIE, 2005). As a consequence, health education *per se* has received little attention and only in few studies been assessed scientifically with proper evaluation of efficacy, effectiveness and impact (WHO, 2015). Recently a computer-based tool, The Vicious Worm, was developed with the hope to upgrade health education to the status of a specific control tool in line with drugs and vaccines (Johansen *et al.* 2014). The tool is a freeware (http://www.theviciousworm.org) and also available as an app (for Android and IOS). It targets stakeholders across disciplines and sectors, providing information about transmission, diagnosis, risk factors, prevention and control of the diseases.

Modelling the effect of health education by reducing the man-to-pig and pig-to-man transmission rates requires the assumption that people will change their risky practices following the education. In the example provided in Fig. 1E, it is assumed that following health education, people will reduce their consumption of infected pork or cook it, infected pork will to a greater extend be condemned, open defecation will be reduced, more safe water will be used, and pigs will be better confined. All together this has been set to reduce the transmission rates by 50% (arbitrary set due to lack of information) over a 3-month period only. In reality, the health education is expected to have a much more prolonged effect especially if repeated annually, but this remains to be elucidated.

### Improved meat inspection, hygiene and sanitation

Control of TSTC in Europe and North America was facilitated through industrialization including meat inspection as well as improved hygiene and sanitation. As a long-term goal, this would be the most sustainable solution but for now unfeasible in most endemic settings. The effect of improving meat inspection and, hygiene and sanitation are included in the health education model despite the lack of studies assessing the efficacy of these measures. A recent study from Zambia failed to show any effect of a Community-Led Total Sanitation programme on the prevalence of porcine cysticercosis in the communities (Bulaya *et al*. 2015).

### Integrated cross-sectoral approach to control

Implementation of combined interventions to prevent and control TSTC has been recommended considering the strengths and limitations of each of the individual control strategies (FAO and WHO, 2014). Nevertheless, currently there is limited information on optimal combinations for cost-effective prevention and control of TSCT in an endemic situation. The final proposed scenario is a 4-year programme combining annual MDA to school-aged children (90% coverage and 100% efficacy) and annual health education at the community level (50% transmission risk reduction in a 3-month period) with vaccination and treatment of pigs aged 1–9 months of age every 6 months for 4 years (100% efficacious vaccine, 90% efficacious treatment and coverage of 75% of the age group 1–9 months which represents 80% of the pig population). Using this integrated approach targeting the parasite in both man and pigs simultaneously, the effect will be highly significant, but will not be able to eliminate the infection in neither man nor pigs (Fig. 1F).

## DISCUSSION

All scenarios were able to reduce taeniosis by at least 50%, but maintaining and further reducing taeniosis is the real challenge. In a study from Tanzania, Braae *et al.* (2015*b*) followed an MDA programme providing praziquantel to school-aged children annually which resulted in a community taeniosis prevalence reduction from 3·0 to 2·0% after the first MDA and to 0·8% after the second MDA. As the project also included track-and-treat of all positive taeniosis cases in the community, the effect cannot only be ascribed to MDA. However, as depicted by the transmission model, no single intervention will be able to control TSTC, but more surprising; a substantial but realistic effort combining the most promising tools over a 4-year period will not result in eliminating the infection in neither man nor pigs. The first goal for the TSTC elimination programmes should therefore be to replace the unrealistic elimination goal with more realistic control goals, and define goals in relation to time. As TSTC is a zoonosis it is important to set the goals for both taeniosis and porcine cysticercosis control and define whether the first target should be morbidity control or transmission control (Bergquist *et al.* 2009). Development of surveillance strategies will also be essential, as the two published *T. solium* models predict fast rises in the prevalence of human taeniosis and porcine cysticercosis once the control programmes end (Gonzalez *et al.* 2002; Kyvsgaard *et al.* 2007).

The model is built assuming a steady human population but having an in- and out-flux of pigs at 3-month intervals. This mimics the situation that most pigs are slaughtered around 1 year of age. In the different scenarios, the prevalence of porcine cysticercosis is affected by the intervention, but as soon as the intervention stops, only very few infected humans will result in a very fast rise in the porcine cysticercosis prevalence as the model assumes that 25% of all pigs are susceptible to *T. solium* at any time point.

There are many limitations in the used theoretical transmission model of which the most important is likely to be the lack of inclusion of the environment. Maya *et al.* (2012) assessed the survival of *T. solium* eggs in waste water and concluded that the eggs survive so well that they should be used as indicator for waste inactivation. As almost no literature exists regarding survival of *T. solium* eggs outside the human host, data obtained in Kenya from the indistinguishable *T. saginata* suggest almost 1 year survival in both dry and wet field conditions (Duthy and van Someren, 1948). Several studies have pointed at ‘water source’ as a risk of *T. solium* transmission, which also underlines the importance of the environment for transmission and the need to design prediction models which includes this factor (Dumontet *et al*. 2001; Maya *et al.* 2012; Mwanjali *et al*. 2013). Another limitation is the lack of an age structure as age of the host has been shown to be important (Braae *et al.* 2014, 2015*b*). As transmission of *T. solium* is socially determined, local human practices may, to a large extend also influence the transmission rates from man-to-pig and from pig-to-man, which is not accommodated in the model (Porphyre *et al*. 2015; Thys *et al*. 2015).

Efficacy of drugs and vaccines is in the current scenarios set unrealistically high. Infrastructure needed to ensure optimal conditions are often insufficient resulting in incorrect storage of drugs and vaccines, lack of measurements for correct dosing, and lack of skilled personnel for correct application, all leading to reduced efficacy in the field.

Compliance is likely to be the main obstacle as people do not recognise taeniosis, hence do not actively seek medical attention. Vaccinating pigs against porcine cysticercosis, which for most farmers is not seen as the most important threat, is likely to have a low compliance (Phiri *et al.* 2003). The best way forward would therefore be to develop an African swine fever vaccine and combine it with the vaccine against porcine cysticercosis. As neither oxfendazole nor porcine cysticercosis vaccines are available in any sub-Saharan country this remains theory. When the tools become available the price will naturally be a main determinant of compliance.

### Concluding remarks

With the ultimate goal to eliminate TSTC, the road is long in SSA. Even the proposed One Health approach with a 4-year strategy did not result in elimination of neither taeniosis nor porcine cysticercosis in a small community. In addition to the theoretical challenges, compliance, cost and logistic challenges will make it very difficult to reach the goal of elimination. The way forward must be a stepwise approach, involving all relevant sectors, setting clear measurable goals for morbidity and transmission control in both man and pigs. A surveillance strategy must be developed and implemented to measure progress and finally, transmission models should continuously be improved and used to guide the way forward.
